# Predictors of neonatal mortality in Ghana: evidence from 2017 Ghana maternal health survey

**DOI:** 10.1186/s12884-023-05877-y

**Published:** 2023-08-02

**Authors:** Emmanuel Ayire Adongo, John Kuumuori Ganle

**Affiliations:** 1 World Child Cancer, Moorfields House, Slater Avenue, Korle Bu, P. O. Box KB273, Accra, Ghana; 2 Ghana College of Nurses and Midwives, 214 Westlands Residential Area, Accra, Ghana; 3 Department of Population, Family and Reproductive Health, School of Public Health, University of Ghana, Legon, Accra Ghana

**Keywords:** Neonatal mortality, Predictors, Antenatal care, Skin-to-skin contact, Ghana

## Abstract

**Background:**

Neonatal mortality contributes about 47% of child mortality globally and over 50% of under-5 deaths in Ghana. There is limited population level analysis done in Ghana on predictors of neonatal mortality.

**Objectives:**

The objective of the study was to examine the predictors of neonatal mortality in Ghana.

**Method:**

This study utilizes secondary data from the 2017 Ghana Maternal Health Survey (GMHS). The GMHS survey focuses on population and household characteristics, health, nutrition, and lifestyle with particular emphasis on topics that affect the lives of newborns and women, including mortality levels, fertility preferences and family planning methods. A total of 10,624 respondents were included in the study after data cleaning. Descriptive statistical techniques were used to describe important background characteristics of the women and Pearson’s Chi-squares (χ^2^) test used to assess association between the outcome (neonatal death) and independent variables. Multivariate logistic regression analysis was done to estimate odd ratios and potential confounders controlled. Confidence level was held at 95%, and a p < 0.05 was considered statistically significant. Data analysis was done using STATA 15.

**Results:**

The prevalence of neonatal mortality was 18 per 1000 live births. ANC attendance, sex of baby, and skin-to-skin contact immediately after birth were predictors of neonatal mortality. Women with at least one ANC visit were less likely to experience neonatal mortality as compared to women with no ANC visit prior to delivery (AOR = 0.11; CI = 0.02–0.56, p = 0.01). Girls were less likely (AOR = 0.68; CI = 0.47–0.98; p = 0.03) to die during the neonatal period as compared to boys. Neonates who were not put skin-to-skin contact immediately after birth were 2.6 times more likely to die within the neonatal period than those who were put skin-to-skin contact immediately after birth (AOR = 2.59; CI = 1.75–3.83, p = 0.00).

**Conclusion:**

Neonatal mortality remains a public health concern in Ghana, with an estimated rate of 18 deaths per 1,000 live births. Maternal and neonatal factors such as the sex of the newborn, the number of antenatal care visits, and skin-to-skin contact between the newborn and mother immediately after birth are the predictors of neonatal mortality in Ghana.

**Supplementary Information:**

The online version contains supplementary material available at 10.1186/s12884-023-05877-y.

## Background

The first 28 days of life are the most delicate period of life for every newborn child [1]. Globally, neonatal mortality constitutes about 47% of child mortality [2]. While global neonatal morality rate was as low as 18 per 1000 live births in 2017, the African Region recorded a high rate of 26.7 per 1000 live births [2] with most of these deaths occurring before the end of the first week of life with about 1 million dying within the first 24 hours and close to one million dying within the next six days [3].

In response to the global and African regional call to action on Reproductive, Maternal, Newborn and Child Health (RMNCH), Ghana signed and committed to the “Every Woman Every Child” and the Global “Newborn Action Plan” [4] as part of efforts to accelerate the reduction of neonatal mortality in Ghana. In 2015, Ghana launched the Every Woman Every Child and the ‘*‘A Promise Renewed’’* as well as the Ghana National Newborn Care Strategy and Action Plan, 2019–2023 [4]. Ghana’s commitment to the SDG target of reducing NMR to at least 12 per 1000 live births by 2030, subscription to the WHO standards of care for small and sick newborns and to the Global Initiative for Quality of Care for Maternal and Newborn [4–6] are among the global commitment to accelerating the SDG NMR target of least 12 per 1000 live births by 2030.

Coverage in maternal and newborn health services have improved in several low-resourced settings including Ghana over the last decade but this did not translate to better outcomes [7, 8]. This is due to several factors including inequitable distribution of human resources, unfriendly health personnel, lack of critical equipment, and poor quality of care [9]. Despite several strategies put in place to reduce neonatal mortality, Ghana is witnessing an increase in institutional neonatal mortality rate with the national figure increasing from 3.8 per 1000 live births in 2014 to 8.4 per 1000 live births in 2017 [10].

Despite Ghana’s commitment, to reducing neonatal morbidity and mortality, neonatal mortality remains an important public health challenge in the country. For instance, Ghana recorded 25 per 1000 live births in 2017 [11], and 27 per 1000 live births in 2017–2018 [12] with about 92% of newborns’ deaths occurring before they reach 7 days [13].

Neonatal mortality is influenced by many factors, including geographical location of parents [13–16]. Several studies also found that a number of socio-demographic factors are strongly associated with neonatal mortality including maternal education, marital status, place of residence, and wealth quintile [13, 14, 16–18]. Neonates whose mothers are affiliated to religious groups have been found to have higher survival probabilities as compared with those whose mothers are without any religious affiliation [16, 19, 20] which is probably due to the accessibility of social support associated with religious groupings. Primiparity has been found to influence neonatal mortality positively in several studies due to the less biological risk therein [20]. In several previous studies, mothers older than forty years had higher neonatal deaths compared with mothers less than forty years of age [21–23]. Also, newborns from wealthier families stand a better chance of surviving in their earlier years than newborns from poorer families [1, 24] due to access to education, health services and social support. In a systematic review and meta-analysis conducted on the impact of antenatal care on neonatal mortality in Sub-Saharan African countries, women who had at least one antenatal care visit had 39% lower risk of neonatal mortality as compared with women with no prior antenatal care visit [25, 26]. However, it has been found in a number of studies that preventive counselling improved maternal and newborn outcomes [27–29]. Women with multiple deliveries have also been found to have higher risk of their newborns dying [21, 30]. Studies have also shown that neonatal mortality is more prevalent in males than females due to several factors including reduced APGAR score, poor foetal development and reduced alveolar epithelial sodium transport [30–34]. Finally, infants with fifth and higher birth orders have higher prevalence of neonatal mortality than infants of lower birth orders [31, 35].

The importance of immediate skin-to-skin contact (SSC) as a protective factor in neonatal mortality cannot be overemphasized [36]. SSC is effective against neonatal mortality and morbidity [37]. In Almgren, 2018, temperature and cardio-respiration showed perfect stability scores in neonates with SSC compared with 46% of conventional care [36]. SSC provides better physiological stabilization to newborns as compared to conventional care [36, 38]. SSC provides the infant warmth, thereby preventing neonatal hypothermia, which is one of the three major causes of neonatal mortality [39]. The effect of SSC in stable neonates is expected to be greater in low-resourced settings where standard care is limited [40].

Although there are existing studies on predictors of neonatal mortality in Sub-Saharan Africa, very little has been done in Ghana. Examples of studies conducted on neonatal mortality in Ghana include Annan and Asiedu [41],, Engmann et al. [42], Kayode et al. [35], and Moyer et al. [43]. While these studies have provided important insights on neonatal mortality in Ghana, they were either based on localized data or old national level data. For instance, Engmann et al. [42] and Avoka et al [44] focused on reasons why babies die in the first month after birth in northern Ghana and in the Eastern Regional Hospital respectively. The Annan & Asiedu’s [41] study on predictors of neonatal deaths in Ghana also focused on a single region -Ashanti Region – using a cross-sectional study data. Afulani’s [45] study focused on whether the quality of antenatal care mattered in determining neonatal death. Similarly, the Kayode et al. [35] multi-level analytical study, described individual and community level predictors of neonatal mortality in Ghana, and used data from the 2003 and 2008 Ghana Demographic and Health Survey. Given the SDG 3 target of 12 neonatal deaths per 1000 live births by 2030, not only is there a need to intensify efforts to further reduce neonatal deaths, but there is also a need for increased research using population-based data to fully understand the causes and predictors of neonatal mortality in Ghana, and the strategies needed to accelerate progress. Ghana requires adequate empirical evidence to support a paradigm shift for an accelerated reduction in neonatal mortality. It is for this reason that this study aimed to examine the predictors of neonatal mortality in Ghana as part of efforts to provide empirical data to support policies and strategies for accelerating the reduction of neonatal mortality in the country.

## Materials and methods

### Study design

This study is an analysis of secondary data from the 2017 Ghana Maternal Health Survey (GMHS). The GMHS survey focuses on population and household characteristics, health, nutrition, and lifestyle with particular emphasis on topics that affect the lives of newborns and women, including mortality levels, fertility preferences and family planning methods [46].

### Study context

The data used for this study covered all the 10 old administrative regions of Ghana. Ghana is in West Africa, just above the Equator with a total population of 30.8 Million according to the 2021 Ghana Population and Housing Census Report [47]. Females constitute approximately 50.7% of the population [47] and the majority of the population (71%) profess Christianity. While the number of people living below the poverty line has declined over the years, conditions for people living in rural regions, especially in the northern part of the country are getting worse with over 60% of people living below the poverty line of GHS 1,314.00 [48]. In terms of healthcare delivery, data from the 2017 District Health Information Management System (DHIMS 2) shows that Ghana had 5 teaching hospitals, 10 regional hospitals, 353 district hospitals, 3 Psychiatric hospitals, 346 Maternity Homes, 38 polyclinics, 1004 health centres, 998 clinics, and 5421 Community-based Health and Planning Service (CHPS) compounds, which provided preventive, curative and rehabilitative services to the population [49]. Notwithstanding the availability of these health facilities, Ghana continues to experience significant challenges in implementing proactive interventions to improve newborn survival.

### Study population

The study population comprised women aged 15–49 years who delivered within 5 years preceding the 2017 GMHS [50]. In terms of inclusion criteria, the data used in the analysis included only responses from women who delivered live babies from 2012 to 2016 (five years preceding the conduct of the 2017 Ghana Maternal Health Survey) and the newborn lived for at least 28 days.

### Sample size

In the 2017 GMHS, 25,062 mothers agreed and provided information on the outcome of their births. A total of 15,371 (61.3%) mothers reported having live births between 2012 and 2016 preceding the 2017 GMHS. However, a total of 10,624(69.1%) records were included in this study after cleaning the dataset. Data elements without responses on the variables reported in this study were treated as missing data and thus, not used in the analysis.

### Sampling method

The GMHS used a multistage sampling procedure in selecting the study participants [11]. The first stage involved simple random sampling of clusters consisting of enumeration areas (EAs) across the country. Overall, 1,900 enumeration areas (EA) were selected − 466 in urban and 434 in rural areas [50]. The second stage of sampling involved a systematic sampling of households within the clusters [50]. A total of 26,324 households within the selected clusters were sampled out of which 25,062 women aged 15–49 were selected [50]. This was done for the then 10 administrative region of Ghana. The sampling frame adopted the 2010 Population and Housing Census (PHC), which contained all enumeration areas (EAs) that covered about 161 households [50].

In this study, the Children’s folder (GHCH7IFL) and women’s folder (GHIQ7IFL) were used as the primary datasets. These two datasets were merged to form one file for the study. A total of 15,371 (61.3%) with live births between 2012 and 2016 were sampled. Out of this number, a total of 10,624(69.1%) sample was included in this study after cleaning the dataset.

### Data source and description

The data used in this study was secondary data and was anonymized. As a result, there was no need for ethical approval. However, permission was sought from the Demographic and Health Surveys (DHS) programme, and permission/access was granted.

The two main data sources for this study were the Household Questionnaire and the Woman’s Questionnaire. The GMHS adapted the Household and Woman’s Questionnaires from the DHS Programme’s Standard Demographic and Health Survey questionnaires used in the 2007 GMHS to reflect the specific interest of the survey [50]. The Household Questionnaire was used to list all members and visitors, take basic demographic information including age, sex, marital status, education, and relationship to head of the household. The Woman’s Questionnaire was used to collect information from women aged 15–49 years. The information collected included background characteristics, pregnancy history (live births, stillbirths, miscarriage, abortion), family planning, pregnancy and postnatal care, abortion, and miscarriage.

### Data processing and management

The Children’s folder (GHCH7IFL) was identified as the primary dataset where most of the variables for this study were found. The individual women folder (GHIQ7IFL) was also identified as a source for variables for maternal education, religion, and wealth quintile. This was merged with the primary dataset (Children’s folder).

To ensure representativeness of the sample in the various clusters and regions, analyses were performed by weighing the samples according to the sampling weight in the 2017 GMHS dataset by using the *‘svy’* command in Stata.

### Study variables

#### Dependent variable

The dependent variable of interest in this study was “age at death (months)” of the most recent deliveries in the last five years preceding the 2017 GMHS. In the GMHS dataset, information on age at death is recorded in days for less than 0–30 days, in months for 1–23 months, and in years for two or higher. For this study, age at death in months was used and categorized into two and the outcome dichotomized into: whether death occurred in age of month zero (i.e., 0–28 days) or between age 1 to 48 months (i.e., 1month − 48 completed months). It was recoded as a binary variable as neonatal mortality, and coded as 0 = if no, and 1 = if yes).

#### Independent variables

A number of variables were identified in the 2017 GMHS dataset as independent variables. Socio-demographic factors included:

1. Maternal age: This is captured in the 2017 GMHS as a continuous variable from 15 to 49 years. For this study, maternal age was recoded as 15–19 years = 1, 20-24years = 2, 25–29 years = 3, 30–34 years = 4, 35–39 years = 5, 40–44 years = 6, 45–49 years = 7.
2. Maternal religion: This was originally recorded as Catholic, Anglican, Methodist, Presbyterian, Pentecostal/charismatic, other Christians, Islam, traditional/spiritualist, no religion, and other. For this study, maternal religion was recoded adding all Christians (Catholic, Methodist, Presbyterian, Pentecostal/charismatic, other Christians) as Christians = 1, Islam = 2, Traditional/spiritualist = traditional = 3, no religion = Atheist = 4.
3. Maternal education: This was recorded in the 2017 GMHS as primary, middle school, Junior Secondary School/Junior High School, secondary/technical/vocational/community, Senior Secondary School/Senior High School/technical/vocational/community, higher. For this study, maternal education was recoded as 1 = no formal education, 2 = Primary, 3 = Junior Secondary School/Junior High School/Middle, 4 = Senior Secondary School/Senior High School/Vocational, 5 = Tertiary.
4. Wealth index: The level of household income was defined using wealth quintile as a proxy. This was derived from household ownership of assets and goods such as radio sets, television sets and refrigerator, dwelling characteristics, type of source of drinking water, toilet facilities, electricity, wall, and floor materials of the house, cooking fuel and means of transport. Each of these assets was assigned a weight generated using principal component analysis and the resulting scores standardized in relation to a normal distribution with a mean of zero and standard deviation of one. Each household was then given a score for each asset and these asset scores were then summed up for and divided into quintiles from lowest (1) to highest (5) [50]. In this study, these categorizations were maintained. Thus, lowest = 1, second = 2, middle = 3, fourth = 4, highest = 5.

Maternal factors examined included:

1. Number of Antenatal Care (ANC) visits: This was originally recorded in the 2017 GMHS as continuous variable, from 1 to 20 visits during pregnancy and “don’t know”. For this study, number of women who had no ANC visits was recoded as 0 = No ANC visit, 1 = 1–4 ANC visits, 2 = > 4 ANC visits. The variable “don’t know” was dropped for the sake of clarity of analysis and interpretation.
2. Place of delivery was originally recorded as her home, other home, public (i.e., government hospital, public: government health post/CHPS), public (i.e., mobile clinic/outreach, public: other public sector, private: hospital/clinic), private (i.e., FP/PPAG clinic), private (i.e., mobile clinic/outreach), private (i.e. maternity home), private (i.e. other private medical centre), and other. For the purpose of this study, place of delivery was recoded as her home and other home as Home delivery, public: government hospital, public: government health post/CHPS, public: mobile clinic/outreach, public: other public sector as Public Health Facility, private: hospital/clinic, private: FP/PPAG clinic, private: mobile clinic/outreach, private: maternity home, private: other private med centre as Private Health Facilities, and other as Others.
3. Mode of Delivery was originally recorded as delivery by Caesarean Section (C/S) and recoded as Yes and No. For this study, delivery by Caesarean Section was renamed as mode of delivery and recorded as Yes as C/S, No as Not caesarean Section.

Newborn factors that were also examined included:

1. Size of child at birth was recorded in the 2017 GMHS as very large, larger than average, average, smaller than average, very small and don’t know. For this study, size of child at birth was recoded as very large and larger than average (> 2.5 kg) as large = 1, average (2.5 kg) as average = 2 and smaller than average and very small (< 2.5 kg) as small = 3. The variable “don’t know” was dropped to provide clarity in the data analysis.
2. Sex of baby was recorded in the 2017 GMHS as girl and boy. This was maintained in this study.
3. Skin-to-skin contact immediately after birth (i.e., kangaroo mother care) was recorded in the 2017 GMHS as skin-to-skin contact immediately after birth, yes, no and don’t know. This was recoded for the purpose of this study as 1 = yes, 2 = no. The variable “don’t know” was dropped to provide clarity in the data analysis.

Datasets with responses of ‘’don’t know’’ were not used in this analysis.

### Data analysis

Descriptive statistical techniques (i.e., frequency and percentage distribution) were used to describe important background characteristics of the women included in the study. In addition, Pearson’s Chi-squares (χ^2^) test was used to assess association between the outcome (neonatal death) and the independent variables. Multivariate logistic regression analysis was also done to estimate the odds ratio and control for potential confounders. Confidence level was held at 95%, and a p < 0.05 was considered statistically significant. All the data analysis was performed using STATA 15.

### Quality assurance measures

The dataset was examined thoroughly, and only relevant information was extracted. We first ran frequencies and percentages on all variables to identify patterns and missing data. Variables with missing data and small frequencies for some categories were recoded. The two authors of this paper independently did the recoding. Comparisons were then made, and where there were any differences, a reconciliation was done before analysis. Finally, data quality was ensured by using Stata issued-commands to recode variables into new categories appropriate for the study. A ‘Stata Do’ file was used throughout the analysis to record all statistical analysis performed. Appropriate changes were made and saved to the Stata Do file when needed after which results were rerun. This process minimised inconsistencies and ensured data quality.

## Results

### Background characteristics of respondents

Most (24.6%) of the respondents were aged 25–29 years and residing in rural areas (51.7%) (Table 1). Over a fifth (24%) (Table 1) of respondents had no formal education, with less than a tenth (6.2%) having tertiary education and most of them being Christians (77.2%) (Table 1). Respondents who did not attend antenatal care (ANC) prior to delivery were 2.3% (Table 1) while 81.3% (Table 1) attended antenatal care more than 4 times during their most recent pregnancy. More than half (67.2%) (Table 2) delivered in public health facilities, with 11.4% (Table 2) delivering in private health facilities and 20.3% (Table 2) had home deliveries. (Table 2). Also, 15.8% had caesarean section deliveries (Table 2).

**Table 1:** Background characteristics of women who had newborn babies in the last 5 years preceding the 2017 GMHS (*n* = 10,624)

Characteristics	Number	Percentage %
**Zone of Residence**		
Southern	4787	45.1
Middle	4098	38.6
Northern	1739	16.3
**Place of Residence**		
Urban	5130	48.3
Rural	5495	51.7
**Maternal Age (years)**		
15–19	564	5
20–24	1928	18
25–29	2612	24.6
30–34	2498	23.5
35–39	1876	17.7
40–44	849	8
45–49	297	2.8
**Maternal Religion**		
Christian	8207	77.2
Islam	1851	17.4
Traditional	250	2.3
Atheist	316	3
**Maternal Education**		
No Formal Education	2552	24
Primary	1875	17.6
JSS/JHS	4209	39.6
SHS/SSS	1330	12.5
Tertiary	658	6.2
**Wealth Index**		
Lowest	2286	21.5
Second	2304	21.7
Middle	2112	20
Fourth	2097	20
Highest	1825	17.2
**Number of ANC visits**		
None	241	2.3
1	141	1.3
2–4	1608	15.1
> 4	8634	81.3

Source: GMHS 2017

**Table 2:** Maternal and Newborn Predictors (%)

Characteristics	Number	Percentage %
**Place of Delivery**		
Home	2159	20.3
Public Health Facility	7135	67.2
Private Health Facility	1208	11.4
Others	122	1.1
**Mode of Delivery**		
Caesarean Section	1277	15.8
Not Caesarean Section	9347	84
**Size of Baby at birth**		
Large	4437	41.2
Average	4264	40.1
Small	1923	18.1
**Sex of baby**		
Boy	5375	50.6
Girl	5249	49.4
**Skin-to-skin contact immediately after birth**		
Yes	5254	49.5
No	5370	50.4

Source: GMHS 2017

### Prevalence of neonatal mortality

Out of the 10,624 respondents who reported to have delivered livebirths within the 5 years preceding the 2017 GMHS, 190 newborns died within the first 28 days of life. This represents a neonatal mortality prevalence of 1.8% or 18/1000 livebirths.

### Predictors of neonatal mortality (bivariate analysis)

Table 3 presents bivariate analysis examining the relationship between neonatal mortality and community level, socio-demographic, maternal and neonatal factors. None of the community level factors were associated with neonatal mortality: Zone of residence (p = 0.26), and place of residence (p = 0.51). Similarly, none of the socio-demographic factors examined showed statistical association with neonatal mortality: maternal age (p = 0.93); maternal religion (p = 0.70); maternal education (p = 0.34); and wealth index (p = 0.81). Among three maternal variables examined, number of ANC visits (p < 0.001) was a statistically significant predictor of neonatal mortality but place of delivery (p = 0.80), and mode of delivery (p = 0.25) were not statistically significant predictors of neonatal mortality. Out of the three neonatal variables examined, sex of the baby (p = 0.03) and skin-to-skin contact immediately after birth (p = 0.00) were statistically significant predictors of neonatal mortality, but size of the baby was not a statistically significant (p = 0.07) predictor of neonatal mortality.

**Table 3:** Predictors of neonatal death (bivariate analysis) *(n = 10,624)*

Characteristics	Neonatal Mortality		P-value
	No, n (%)	Yes, n (%)	
Place of Residence			
Urban	5044 (98.3)	86 (1.7)	0.51
Rural	5390 (98.1)	104(1.9)	
Zone of settlement			
Southern	4690(98)	97(2.0)	0.26
Middle	4029(98.3)	69(1.7)	
Northern	1715(98.6)	24(1.4)	
Maternal Age (completed years)			
15–19	552(97.9)	12(2.1)	0.93
20–24	1898(98.5)	30(1.5)	
25–29	2571(98.4)	41(1.6)	
30–34	2449(98.1)	49(1.9)	
35–39	1840(98.1)	36(2.1)	
40–44	832(98)	17(2)	
45–49	291(98.2)	6(1.8)	
Maternal Religion			
Christian	8060(98.2)	147(1.8)	0.70
Islam	1819(98.3)	32(1.7)	
Traditional	247(98.7)	3(1.2)	
Atheist	307(97.2)	9(2.8)	
Maternal Education			
No Formal Education	2514(98.5)	38(1.5)	0.34
Primary	1836(97.9)	39(2.1)	
JHS/JSS/Middle	4136(98.3)	73(1.7)	
SHS/SSS/Vocational	1295(97.4)	35(2.7)	
Tertiary	651(99.0)	7(1.0)	
Wealth Index			
Lowest	2242(98.1)	44(1.9)	0.81
Second	2263(98.2)	41(1.8)	
Middle	2072(98.1)	40(1.9)	
Fourth	2056(98.0)	41(1.9)	
Highest	1801(98.7)	24(1.3)	
Number of ANC visits			
None	227(94.2)	14(5.8)	0.00**
1	140(99.3)	1(0.7)	
2–4	1579(98.2)	29(1.8)	
> 4	8487(98.3)	147(1.7)	
Place of Delivery			
Home	2117(98.1)	42(1.9)	0.80
Public Health	7005(98.2)	130(1.8)	
Private Health	1191(98.6)	17(1.4)	
Other	120(98.6)	2(1.4)	
Mode of Delivery			
Caesarean Section	1247(97.6)	30(2.3)	0.25
Not Caesarean Section	9187(98.3)	160(1.7)	
Sex of baby			
Boy	5261(97.9)	114(2.1)	0.03*
Girl	5173(98.6)	76(1.5)	
Size of baby at birth			
Large	4360(98.3)	78(1.7)	0.07
Average	4192(98.3)	72(1.7)	
Small	1881(97.8)	41(2.2)	
Skin-to-skin contact immediately after birth			
Yes	5200(98.9)	54(1.0)	0.00**
No	5234(97.5)	136(2.5)	

Source: GMHS 2017,

*P < 0.05; **p < 0.01

Sex of the newborn, number of antenatal (ANC) attendance and baby put on mother’s chest immediately after birth) were statistically associated with neonatal mortality (Table 4). To further determine the strength of these variables, confounders were controlled in a multiple regression model and adjusted odd ratios were estimated.

The odds of a woman who had at least 1 ANC visit prior to delivery experiencing neonatal mortality was significantly lower compared to those who had no ANC visit prior to delivery (aOR = 0.11; CI = 0.02–0.56, p = 0.01) (Table 4). Women with 2–4 ANC visits were also less likely to experience neonatal mortality as compared to women who had no antenatal care (aOR = 0.29; CI = 0.12–0.68; p = 0.01) (Table 4). Also, women with more than 4 antenatal visits were less likely to experience neonatal mortality as compared to women with no antenatal visits prior to delivery (aOR = 0.29; CI = 0.13–0.67; p = 0.00) when potential confounders were controlled (Table 4).

In a binary logistic regression, girls were less likely to die during the neonatal period as compared to boys and this did not change when potential confounders were controlled for in a multiple logistic regression model (aOR = 0.68, CI = 0.47–0.98; p = 0.04). Similarly, the odds of a baby dying within the neonatal period when the baby was not put in skin-to-skin contact immediately after birth was 2.6 times higher than those put skin-to-skin contact immediately after birth (aOR = 2.59; CI = 1.75–3.83; p = 0.00) (Table 4).

**Table 4:** Predictors of neonatal mortality (*Multilevel Regression Analysis*), **n = 10,624**

Characteristics	Neonatal Mortality		P-value	aOR[95%CI]	P-value		
	No, n (%)	Yes n (%)					
Number of ANC visits							
None (Ref)			1				
1	140(99.3)	1(0.7)	0.01*	0.11(0.02–0.56)	0.01*		
2–4	1579(98.2)	29(1.8)	0.00**	0.29(0.12–0.68)	0.01*		
> 4	8487(98.3)	147(1.7)	0.00**	0.29(0.13–0.67)	0.00		
Sex of baby							
Boy (Ref)	5261(97.9)	114(2.1)	1				
Girl	5173(98.6)	76(1.5)	0.03*	0.68(0.47–0.98)	0.04*		
Skin-to-skin contact immediately after birth							
Yes (Ref)	5200(98.9)	54(1.0)	1				
No	5234(97.5)	136(2.5)	0.00**	2.59(1.75–3.83)	0.00**		

Source: GMHS 2017

aOR = adjusted odds ratio; CI = confidence interval; ref = reference category; *p < 0.05; **p < 0.01

## Discussion

This study sought to find out the predictors of neonatal mortality in Ghana.

We found out that the prevalence of neonatal mortality was 1.8% (18 per 1000 live births). This is lower than the 2017 GMHS [50] rate of 25 per 1000 live births and by the Multiple Indicator Cluster Survey (MICS), 2017–2018 [51] results. This is because the original sample size was cleaned and weighted using Stata. Also, the sample size used was only limited to live births. Respondents who had live births with responses ‘don’t know’ or had no responses (empty) for the indicators used in this study were not used in this analysis and this reduced the sample size from 15,371 to 10, 624 (69.1%). However, 18 per 1000 live births is still higher than the SDG 3 target of 12 per 1000 live births by 2030 [52]. Ironically, facility delivery increased from 54% to 2007 to 79% in 2017, an increase of 32% which was expected to translate to better maternal and newborn outcomes but this has not been the case [12, 53]. This is probably due to inadequate facility readiness for the care of low-risk, normal -weight/term newborns, and at-risk/small, and sick newborns [4]. Ghana needs to adopt evidence-based solutions and cost-effective interventions that have been proven and tested to reduce neonatal mortality in LMICs and these include but are not limited to skin-to-skin contact immediately after birth, breastfeeding, Focussed Antenatal Care, and administration of antenatal corticosteroids for women in preterm labour [54]. Ghana’s paradigm shift to focus on quality of newborn care at the facility level and promote holistic care for newborns enshrined in the Ghana National Newborn Care Strategy and Action Plan, 2019–2023 is commendable if the strategy will be followed to the letter and not business as usual [4].

Maternal and neonatal factors including number of antenatal visits, sex of newborn, and skin-to-skin contact immediately after birth have been found to be predictors of neonatal mortality. In both bivariate and multivariate analysis, we found out that the more often a woman attends antenatal clinic for routine checkups, the more protected she is against neonatal mortality. This is consistent with findings by Tekelab et al., 2019 where only one antenatal visit reduces the risk of neonatal mortality by 39% in Sub Saharan Africa [55]. Women who received any ANC care increased from 96% to 2007 to 98% in 2017 but this unfortunately did not translate to positive outcomes, and this could be associated with the quality of antenatal care [7, 45]. Thus, while the number of antenatal visits made may not necessarily result in improved maternal and neonatal outcomes because of the quality of the service package [45], ANC attendance with preventive counseling may ensure that risk factors are identified and resolved early to improve maternal and foetal outcomes [28, 56].

We found sex of newborn to be a statistically significant predictor of neonatal mortality in both bivariate and multivariate analysis. In our study, females had 32% lower odds of dying during the neonatal period as compared to their male counterparts. This is consistent with findings from Muhammed et al., 2018 [33] and Engmann et al., 2012 [57]. This is due to the biological advantage of females having better survival rates compared to their male counterparts [34, 58]. These biological factors are particularly associated with the slow development and maturity of male infant lungs compared to their female counterparts [32, 58]. This has been confirmed by scientists. In the United Kingdom they found male neonates having lower mean APGAR scores at 5 min, experiencing more episodes of apnoea, and bradycardia, having transient tachypnoea, and pneumonia more often than female neonates [59]. Ghana can take advantage of these available pieces of evidence to support strict implementation of the Ghana National Newborn Strategy and Action plan 2019–2023 which entreats the Ministry of Health and its agencies to develop/strengthen/ update policies and tools to promote optimal follow-up care of all newborns including the at-risk, small, and sick babies, including neurodevelopmental assessment and ECD activities [4].

In our bivariate and multivariate analysis, we found that newborns who did not receive immediate skin-to-skin contact (SSC) were 2.6 times more likely to die compared with those who did. This is confirmed by Moore et al., 2016 in a systematic review and Almgren, 2018 where it was reported that neonates who experienced SSC immediately after birth had better physiological stabilization [36, 38]. SSC provides the infant warmth, thereby preventing neonatal hypothermia, which is one of the major causes of neonatal mortality [39].

Our study is limited by only data from women with live births, neonates were from age zero to 28 months in the Ghana Maternal Health Survey 2017 data extracted as well as those with adequate responses. Also, respondents with ‘’don’t know’’ and empty responses, were not used in the analysis. However, the strength of the study is that it is national in character as population-based survey data was used for the analysis.

The findings of this study point to inadequate antepartum, intrapartum, and postpartum quality of care. The gap in service quality needs to be addressed from antenatal care, through intrapartum and postnatal periods to ensure that newborns survive and thrive.

## Conclusion

The predictors of neonatal mortality are maternal and neonatal factors (i.e., sex of baby and number of antenatal visits prior to delivery, and skin-to-skin contact immediately after birth) in Ghana.

Neonatal mortality remains a public health concern as coverage of maternal and newborn health service is not translating to better outcomes. The Ministry of Health and its agencies including Ghana Health Service should not do business as usual but implement the Ghana National Newborn Strategy and Action Plan, 2016 to 2023 to the letter. The policies and strategies of the country are apt and consistent with recent global and regional policies and strategies. What is required is to focus on quality of care at all levels using tools already developed to address the quality gaps in service delivery.

## Limitations of the study

The data extracted was limited to those with live births from age zero to 48 months old. Also, respondents with ‘’don’t know’’ responses for the indicators used for this study were not included in the final analysis.

Limitations and challenges of the DHS system also affected the results of this study. These include errors such as recall bias and social desirability responses.

## Electronic supplementary material

Below is the link to the electronic supplementary material.

Supplementary Material 1

## Data Availability

The dataset used in this article is from the dhsprogramme and can be accessed upon request to the administrator. The link to the primary dataset used in this study can be accessed via https://dhsprogram.com/data/dataset_admin/index.cfm.

## List of abbreviations

ANC: Antenatal Care
aOR: adjusted Odds Ratio
CHPS: Community-based Health and Planning Service)
CI: Confidence Interval
cOR: crude Odds Ratios
DHIMS: District Health Information Management System
DHS: Demographic and Health Survey
ENC: Essential Newborn Care
GHS: Ghana Health Service
GSS: Ghana Statistical Service
GMHS: Ghana Maternal Health Survey
LMICs: Low- and Middle-Income Countries
MoH: Ministry of Health
PAS: Public Address System
RMNCH: Reproductive, Maternal, Newborn and Child Health
SBCC: Social and Behaviour Change Communication
SDG: Sustainable Development Goal
SSC: Skin-to-Skin Contact
WHO: World Health Organization
